# An Ecological Perspective of Food Choice and Eating Autonomy Among Adolescents

**DOI:** 10.3389/fpsyg.2021.654139

**Published:** 2021-04-21

**Authors:** Amanda M. Ziegler, Christina M. Kasprzak, Tegan H. Mansouri, Arturo M. Gregory, Rachel A. Barich, Lori A. Hatzinger, Lucia A. Leone, Jennifer L. Temple

**Affiliations:** ^1^Nutrition and Health Research Lab, University at Buffalo, Buffalo, NY, United States; ^2^Department of Exercise and Nutrition Sciences, University at Buffalo, Buffalo, NY, United States; ^3^Department of Community Health and Health Behavior, University at Buffalo, Buffalo, NY, United States; ^4^Community Health Interventions Lab, University at Buffalo, Buffalo, NY, United States

**Keywords:** adolescence, autonomy, eating behavior, food choice, food environment, pediatrics

## Abstract

Adolescence is an important developmental period marked by a transition from primarily parental-controlled eating to self-directed and peer-influenced eating. During this period, adolescents gain autonomy over their individual food choices and eating behavior in general. While parent-feeding practices have been shown to influence eating behaviors in children, little is known about how these relationships track across adolescent development as autonomy expands. The purpose of this qualitative study was to identify factors that impact food decisions and eating autonomy among adolescents. Using the food choice process model as a guide, four focus groups were conducted with 34 adolescents. Focus group discussion was semi-structured, asking teens about influences on their food choices across different food environments, their involvement with food purchasing and preparation, and perceived control over food their choices. Focus group transcripts were analyzed using deductive and inductive code creation and thematic analysis. This study found six leading influences on adolescents' food choices and identified additional factors with prominence within specific environmental contexts. This study distinguished a broader spectrum of factors influencing adolescent food choice that extend beyond “convenience” and “taste” which have previously been identified as significant contributors. The degree of control that teens reported differed by eating location, occasion, and social context. Finally, adolescents demonstrated various levels of engagement in behaviors related to their eating autonomy. Identifying the emergent themes related to adolescent autonomy was the first step toward the goal of developing a scale to evaluate adolescent eating autonomy.

## Introduction

Adolescence is an important developmental period marked by a transition from primarily parental-controlled eating to more self-directed eating (Kelder, 1994). Adolescence spans the period from ages 10–19 and is characterized by rapid physical, mental, and emotional development (Sawyer et al., 2018). During this life stage, adolescents' food choices and eating are influenced by parents, peers, and the surrounding environment (Story et al., 2002). Since studies have shown that the food choice behaviors of children and adolescents track into adulthood, it is valuable to gain a better understanding of factors that influence adolescent eating behavior (Devine, 2005). In addition, the degree to which an adolescent has control, or autonomy, related to food choices may be associated with other health outcomes, such as eating disorders or obesity.

Much of the prior work in adolescents has focused on disordered eating behaviors, eating in specific contexts, or types of foods. For example, there is a large body of evidence surrounding maladaptive adolescent behaviors such as meal skipping (Nicklas et al., 2001), extreme dieting, (Patton et al., 1997), and binge eating (Swanson et al., 2011; Pearson et al., 2012). There is also a robust literature showing that food parenting practices and parenting styles may influence the eating behaviors of offspring, but less is known about the effects on adolescents specifically (Savage et al., 2007; Vaughn et al., 2016). Age-based characteristics, such as higher susceptibility to peer pressure, have also been found to impact adolescent food perceptions and choices, primarily in the school environment (Maxwell, 2002; Andersen et al., 2016; Macchi et al., 2017). Finally, much of the work examining adolescents' food choice process has been limited to a sub-set of the diet, such as making “healthy” food choices (French et al., 2001) despite consistent findings showing that teens do not follow healthy eating recommendations (Croll et al., 2001). More research is needed that broadly examines adolescent eating behavior across a variety of eating contexts and eating occasions.

**Figure 1:**
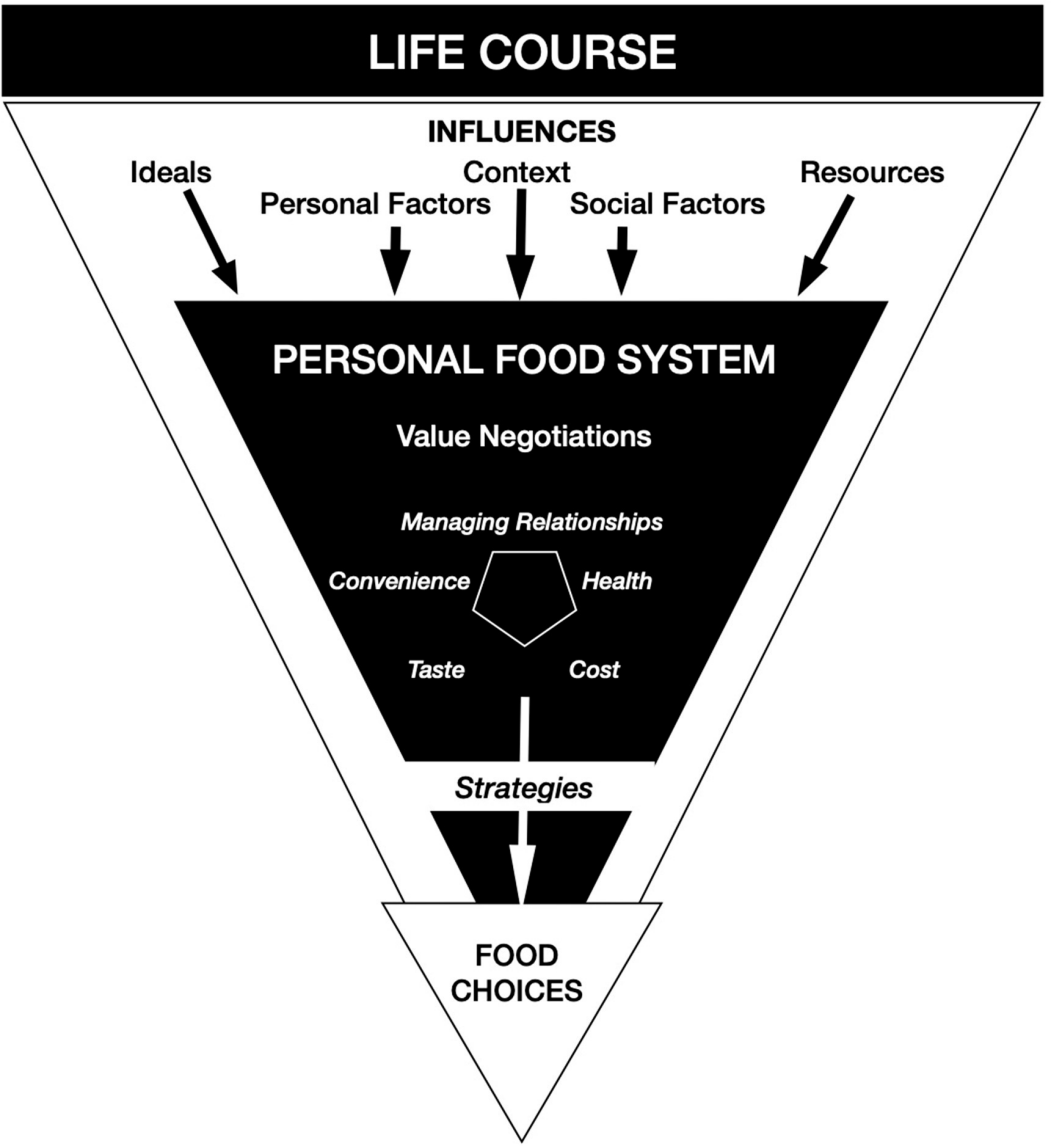
The food-choice process model adapted from Connors et al. (2001).

The present study is informed by several models and theories. The food choice process model provides a framework for examining the complex nature of food decision making across the life course (Sobal et al., 2006). This model, adapted from Connors et al. (2001), was developed based on qualitative investigations of the food choice process in adults; acknowledging that there is individual variability in factors involved in the food choice process (Figure 1). One of the main components of the model is “personal food system” which is the operationalization of perceived influences on food choices across various contexts (Connors et al., 2001). Identifying the factors that an individual has agency over helps to describe active considerations and social negotiations related to making food choices. The food choice process model further explains that within the personal food system, these factors often compete for prioritization based on their value in each context. Expansions of this model have included food behaviors (such as acquisition, preparation, and consumption) produced through food choice decisions (Bisogni and Sobal, 2009). There are important differences between how adults and adolescents conceptualize factors involved in making food choices, but more work is needed to understand the adolescent perspective (Share and Stewart-Knox, 2012).

However, no single theory completely explains food decision making behavior and the food choice process (Bisogni and Sobal, 2009). While, the food choice process model illuminates the importance of understanding adolescents' perceptions of the factors that influence their food choices, some other models heavily emphasize social and environmental influences on eating behavior. Story et al. (2002) proposed a conceptual model of adolescent food choice based on a combination of Social Cognitive Theory (SCT) and the Social Ecological Model (SEM) (Story et al., 2002). This model highlights the importance of examining interactions across the main areas of influence (individual/intrapersonal, social environment/interpersonal, physical environment/community, and macrosystem/societal) within an individuals' food choices.

There have been limited analyses of factors that impact adolescent food choice. Some recent qualitative investigations examined school lunch food choices (Contento et al., 2006; Waddingham et al., 2018). Of those, one asked teens to compare their lunch decisions to other meals or times of day (Contento et al., 2006). Other studies used a recall activity to help adolescents describe the food choices they had made the previous day; one within a school-based, focus group setting (Neumark-Sztainer et al., 1999), and the other through a focus group and individual interviews (Sommer et al., 2014). There has also been at least one study assessing adolescents' food choice process within the home environment (Holsten et al., 2012). More work needs to be done to understand the environmental and psychosocial factors that impact adolescent food choices. To our knowledge, there has not been a qualitative assessment of adolescents' food choices conducted outside of the school or home settings.

Children and adolescents are increasingly recognized as having active roles in their experiences and holding unique perspectives which should be investigated firsthand (Smith, 2007). Understanding the adolescent perspective is particularly important during the life stage where autonomy development occurs across many contexts, including food choice and eating behavior (Spear and Kulbok, 2004; Stok et al., 2010; Dahl et al., 2018). The psychology and development literature suggest that an adolescent's autonomy is related to substance use behavior (Allen et al., 2012), susceptibility to peer pressure (Allen et al., 2006), and self-esteem (Allen et al., 1994). Importantly, a conceptual analysis of the previous adolescent autonomy literature highlights a vacancy in studies examining autonomy relationships with adolescent lifestyle behaviors, such as food choices (Spear and Kulbok, 2004). An association between adolescent autonomy and unhealthy snack purchasing has been identified (Stok et al., 2010), but no other work has been done in this area. Understanding adolescent autonomy related to eating behaviors may be key for tailoring adolescent eating behavior interventions toward clinically significant improvements in dietary and health outcomes.

When adolescents act autonomously, they take on a level of responsibility for and control over their food choices (Teixeira et al., 2011). The terms “autonomy” and “independence” can both reflect freedom from external control, but “independence” can also be used to convey physical separation. Therefore, throughout this work we have defined autonomous food choices as those made without direct parental guidance, using the term “independent” to communicate instances of food choices when adolescents are apart from parents.

To date there is no established way of assessing adolescents' autonomy within the realm of food choice and eating behavior. We have operationalized adolescent eating autonomy as an adolescents' individual decision making related to, and perceptions of control over, their food choices. The primary aim of this study was to identify the predominant factors involved in adolescents' food choices across a variety of ecological environments and social contexts. The secondary aim of this work was to identify the themes related to the perceived control that adolescents' have over their eating. This formative work is a first step toward developing a scale to assess adolescent eating autonomy.

## Materials and Methods

This study used qualitative methods to gain an understanding of adolescents choices related to food. Four focus groups were conducted with adolescents aged 13–17 years between November and December 2019. Discussion focused on exploring the decisions adolescents make, and perceived control, around their food choices.

### Focus Group Recruitment and Setting

Adolescents were recruited via physical and digital flyers distributed to multiple school-districts and list-serves in the Western New York area from October through November 2019. Using convenience sampling, parent(s)/guardian(s) [hereafter referred to as parent(s)] of interested teens were invited to contact the research lab for further information and to complete an eligibility screening. Parents provided demographic information during the screening process. Adolescents were eligible if they were between 13 and 17 years of age, spoke English, and were not concurrently enrolled in another study by our research team. Enrollment was structured to obtain a representative sample of adolescents from different geographic backgrounds (i.e., from urban, suburban, and rural areas). The recruitment coordinator (AMG) used elements of purposive selection to balance scheduled participation in each focus group as evenly as possible based on sex, age, and school to maximize heterogeneity within each focus group sample.

Focus groups were conveniently located based on participant preference. Each focus group followed a near identical flow of events and lasted 1.5 h. Upon arrival to each focus group, teens received an identification number and selected a first name (allowing for anonymity). Then the participant and their parent were guided to a semi-private area where a trained research assistant performed a written parental consent and adolescent assent process. After signing these documents, families were offered a self-serve taco bar dinner to increase rapport with participants and decrease the time burden to participants during the weeknight. After eating, parents left the room and the food was removed until after the focus group finished. Upon completing the discussion questions, teens completed a brief survey packet and received cash compensation for their participation. All procedures were approved by the University at Buffalo Institutional Review Board.

### Discussion Guide

Focus groups followed a semi-structured discussion guide. Participants were told that “we want to better understand how teens decide on the foods that they eat and where they get them.” The discussion questions were designed to be open ended, covering topics such as how teens decide what to eat in a variety of settings, participation in the grocery shopping process, and identifying how and when teens feel the most control over their food choices. The discussion moderator guide, seen in Table 1, laid out priority questions with possible follow-up prompts, to stimulate a conversation that was naturalistic, detailed, and informative about the individual and environmental factors that may play a role in adolescent food choices.

**Table 1:** Focus group discussion guide questions and probes.

**Question**	***Probe***
What are some of your favorite foods?	*How often do you eat them?*
What types of snack foods do you like best?^*^	*Where do you usually get snacks?*
How do you feel about the food options in your house?	*at school?*
How do you choose what to eat when you need to pick something out yourself?	*What things/factors are important to you when you are choosing what to eat?* *Do you have more choices over certain meals? -Times of day? How would you describe your process of picking food after school?*
How much input do you have in grocery shopping?	*Do you help make lists or go along shopping? Some other way?* *How often do you get the things you want?*
What things do you have independence/ control over with your eating?	*Certain meals? Times of day? Locations?*
How do you make meal choices when you are not with your parent/caregivers?	*What motivates you toward certain foods?* *Do you spend your own money on foods/snacks?*
What makes your food choices different than your parent/guardians?	*-different than siblings? Peers?*
If it was up to your parents or parent/caregivers would you eat differently?^*^	*What would they like you to eat more of? - less of?*
How much do your parents/caregivers know about what you eat when you are not with them?^*^	*Is there anything you specifically don't tell them? - Why?*
Is there anything you wish was different about how you choose your food?^*^	

* *Represents lower priority questions*.

### Data Analysis

Recordings were transcribed and reviewed for discrepancies by a research assistant not directly involved in the focus group discussion. Transcripts were independently coded by 3 coders using Atlas.ti 8 (Atlas.ti Scientific Software Development, Berlin, Germany) software. The coders were trained by a co-investigator with qualitative data collection and analysis expertise. Coding and analyzing the data followed a multi-pass thematic coding process. The initial round of coding involved a deductive process of open coding by each independent coder (AMZ, CMK, THM) of all 4 focus groups. The coders met to reconcile differences and begin the development of a codebook. The codebook was utilized for a second round of refined, or focused, coding in which the coders met regularly to debrief, refine the codebook, and begin the inductive process of generating overarching themes. Finally, all coders' transcripts were merged into a final project in which the codes were reconciled among coders and predominant themes were identified. The coders determined that code saturation had been reached when coding of additional focus group transcripts produced codebook revisions related to the use of existing codes rather than generating new codes. Meaning saturation was sufficiently captured through 4 focus groups and confirmed by the same predominant themes emerging across the focus group strata (Hennink et al., 2019).

### Sample Characteristics

There were 69 adolescents that were eligible to participate. Thirty-four eligible teens did not participate: 1 did not show up as scheduled, 8 did not respond to contact, 10 declined participation, and 15 were unable to participate due to scheduling conflicts. The final sample included 35 adolescents, 18 males and 17 females, with mean age of 14.9 years. The final size of each of the four focus groups ranged from 6 to 12 participants: Focus group A (*n* = 7; 4F, 3M), Focus group B, (*n* = 10; 5F, 5M), Focus group C (*n* = 12; 6F, 6M), and Focus group D, (*n* = 6; 2F, 4M). The teens represented a wide range of economic, racial/ethnic, and geographic backgrounds. Fifteen adolescents were from high population density areas, 11 from medium, and 9 from low population densities. These teens attended 26 unique schools. Just over 31% of the sample qualified for free or reduced-price school lunch, however the majority of the sample were from middle- and high-income brackets. In terms of racial/ethnic background, 77.1% identified as White/Caucasian and 14.3% as Black/African American, and 8.6% multi-racial. This is representative of the Western New York population under 18 years old; with 79.3% of the population identifying as White/Caucasian, 14% Black/African American, and 5.8% Hispanic (US Census Bureau, 2020).

## Results

The focus group analysis revealed that the predominant factors impacting adolescents' food choices differ across environments (home, school, restaurants, and stores). Additionally, throughout the focus group analysis, attention was paid to uncover thematic areas unique to the adolescent perspective, where adolescents may experience a range of autonomy over their food selections. These emergent themes identified key elements of the adolescent eating autonomy construct.

### Factors in Adolescents' Food Choices

Adolescents identified many factors that impact their food choices across different contexts. The six predominant factors that adolescents considered when making independent food choices include: schedule and time priorities, hunger level and/or satiety, healthfulness, convenience (and ease of preparation), availability, and physical activity. Predominant factors were those mentioned most across focus groups; defined as the factors voiced by 7–10 adolescents. Less frequently mentioned factors in adolescents' food choices (mentioned by 4–6 adolescents) were cost, portion size/amount, daily nutritional balance, openness to new food, variety, and taste. Notably, the most discussed food selections that adolescents make when making independent choices include mixed dishes (such as sandwiches and pizza), crunchy and salty snacks (such as chips and popcorn), fruits and vegetables, and sweets (such as candy and cookies).

### The Socio-Environmental Context of Adolescent Food Choices

Table 2 shows the summary of results surrounding the predominant factors influencing adolescents' food choices organized by environment. Given that this study draws from SCT, SEM, and the food choice process model, we anticipated the home and school to be common environmental contexts for adolescent food choice. Throughout the coding process other locations, such as restaurants and stores, also emerged as environments in which adolescents' often make food choices.

**Table 2:** Summary of factors influencing adolescents' food choices by environment.

**Environment**	**Factor**	**Expanded context**
*Home (n = 13)*	Schedule and time priorities	Time of arrival, proximity to mealtime
	Availability	Prioritized more as hunger increased
	Convenience and ease of preparation	Prepared simple, familiar items
		Interpersonal relationships not often mentioned
*School (n = 16)*	Schedule and time priorities	School lunch consumed because time or energy for packing a lunch is limited
	Convenience	
	Food quality^**^	Selections described as needing improvements in terms of variety, taste, quality, freshness, consistency, or preparation method
	Taste^*^	
	Variety^*^	More variety of options perceived positively
		Interpersonal relationships not often mentioned
*Restaurant (n = 14)*	Convenience	
	Cost^*^	More cost-conscious choices made when dining without family
	Healthfulness	Categorized options as healthy or unhealthy
	Openness to new foods^*^	Use of communal behaviors when dining with peers
*Store (n = 13)*	Convenience	Convenience stores and gas stations were frequent and accessible locations where adolescents described making food choices
	Cost^*^	Selected special items, not normally consumed
		Often with peers
*General Environments^#^* *(n = 3)*	Hunger and satiey	Considered hunger both in food choices made in the present moment and based on anticipated hunger when planning meals/snacks or packing lunch.
	Physical activity	

*Predominant Factors are those that were mentioned by 7–10 adolescents across focus groups. Less dominant factors were mentioned by 4–6 individuals across focus groups*.

*Symbol Key*.

* *reflects emergence of a less dominant factor in this context*.

** *reflects emergence of a factor unique to this context*.

# *This section lists predominant factors generally mentioned without a location context*.

#### Home

About a third of participants described food choices taking place at home. Within the home environment, the leading food choice factors were schedule and time priorities, availability, and convenience. Many teens indicated making decisions based on what time they arrive at home and proximity to mealtime. One teen explained making their food choices based on “*…[the] time I get home from practice and how close it is to dinner, or when I have to leave or something…”* They also described that they would settle for more convenient/available, but less-preferred foods, based on their hunger level.

Adolescents expressed various levels of involvement in household meal planning (*n* = 11). Some adolescents help their families plan meals in advance every week, which serves as an opportunity to request specific items or meals. One teen described, “*My family, we like plan out our meals every Sunday. I mean this is recent so we can decide like what we want to eat….we just say whatever we want and when we say what we want we get it.”* Others indicated that the parent(s) decide the household meals without teen input, which is acceptable to some, but not others.

Adolescents participate in varying degrees of food preparation at home (*n* = 21). About half of participants described that when preparing meals for themselves, they choose something simple that they know how to prepare. They make something quick “*like grilled cheese or mac ‘n cheese*” or items that require minimal preparation “*like frozen chicken fingers or something like frozen veggies.”* Adolescents decide what to prepare based on availability in the home, citing they will make “*whatever is in the kitchen to cook.”* However, despite occasions of parents leaving food preparation instructions while they are away, some teens stated that they choose more convenience foods anyway: “*But when we get home from school, my sister and me, we don't want to make food so we usually just grab chips or something.”* While some adolescents said they do not cook at all, five participants mentioned that they prepare meals for the family as a whole. Apart from one mention of cooking with a sibling, other individuals were not described within the context of impacting adolescents' food choices at home.

Adolescents' engagement in packing school lunches ranged considerably (*n* = 11). In some cases, parents pack an adolescent's lunch without input, while other teens stated that they regularly select some, or all, of their own packed-lunch food items. Those who packed their own lunch explained that packing lunch is not always possible due to time priorities. Packing one's lunch was described as giving teens higher control over their lunch food choices. When packing their lunch, adolescents make food decisions based on item availability and their anticipated hunger level by considering their physical activity schedule. One explained, “*For me, it's usually just like what I have, because normally I expect a sandwich and, like, a side, like, usually it's yogurt for me, because that's just always what we have in our house. So really, I just pack what's, like, available for me. You know?”*

#### School

About half of participants described their food choices related to the school environment. While some teens noted convenience or scheduling considerations as factors related to their food decisions at school, other major factors unique to the school context emerged. Food quality, freshness, doneness, and the taste of the food were leading considerations for adolescents in this environment. Adolescents frequently expressed their perceptions of the school food environment, in relation to variety and the need for improvement. In the majority of cases, teens shared their reasons for choosing *not* to obtain food from school due to the selections needing improvements in terms of variety, taste, quality, freshness, consistency, or preparation method. Another stated, “*I bring my lunch every day because the school food is really nasty.”* Adolescents that positively regarded their school food options also perceived that there was a lot of variety of the offerings, stating satisfaction with the school food options was “*Because at my school, there is a lot to pick from.”*

Others described eating school lunch as a consequence for not having time or energy to pack a lunch. Adolescents also explained that their busy schedules lead them to consume items from the school vending machine between practices/meetings as a snack or as a meal replacement. Interestingly, only two participants mentioned the influence of peers on their choices within the school environment; with one describing trading food items that they buy at school (such as stuffed crust pizza) for “treats” friends bring from home (such as cookies) and the other explaining that they get breakfast at school “*because all of my friends do it and they have cereal and stuff.”*

#### Restaurants

Forty percent of adolescents described making independent food choices in restaurant establishments. The prominent factors that influence food choices in the restaurant setting include cost, healthfulness, openness to trying new foods, and convenience. Discussion of restaurant dining included fast-food/fast-casual establishments, coffee shops, or “restaurants” that were not specified. Of those discussing restaurant food choices, 40% specifically mentioned being in the company of friends when making food choices at various restaurants.

Participants stated that cost and availability of spending money influence the choice of restaurant and food choice when eating independently. Participants suggested that their restaurant food choices were cheaper when eating without family, stating, “*I usually order something, like, cheaper …I would get, like, just like five chicken wings or something instead of, like, what we would order with family.”* Others cited restrictions when dining at restaurants with family and expressed that they have more freedom to “*just get what I want”* when eating among friends. Peers influenced adolescents' food choices through communal behaviors, such as taking turns paying for food and sharing items. However, while some teens were amenable to buying and sharing food, others noted that they would not share with friends if they spent their own money on the items. Additionally, eating at a restaurant with peers was cited as an opportunity to try new foods, “*that's when I try new stuff - when I'm not with my parents or my family. I just… I'm like, oh that looks good. Let me try it.”*

#### Stores

About one third of participants described making independent food choices at stores, with convenience stores (including gas stations) being the primary location, followed by grocery stores, and drug stores. Adolescents' descriptions of food choices at stores were uniquely social compared to other environments. Among participants that mentioned making food choices at a store, 88% included the company of a friend or cousin. The season was specifically mentioned in about 30% of these descriptions, such as “*During the summer I went to NOCO Speedway with my friends sometimes.”* Some mentioned shopping without parents at grocery stores in the context of being able to buy what they want without parental approval. Often teens shop at grocery stores with friends in preparation for a group hang out. “*Whenever me and my friend hang out, we go to a grocery store. It's like the only time I buy food for myself.”* However, when shopping for household groceries, adolescents mentioned sticking to a grocery list.

### Emergent Themes Related to Adolescent Eating Autonomy

#### Food Acquisition

The theme of independently seeking out food (i.e., acquisition) emerged related to adolescent eating autonomy. Forty percent of participants indicated that they coordinated their own transportation to obtain desired food or drink. Walking and public transit were the most often cited means of independent travel. Adolescents walked to drug stores, convenience stores, restaurants, and grocery stores for food purchases. Lesser used modes of transportation include biking, driving, or getting a ride. Biking was mentioned as a means of independent transit, used in the summer and typically with peers. One teen explained: “… *my cousins and I, we rode up to get ice cream and maybe … we got fries. Then, at 7-Eleven, I would normally get like a can of pop [soda/cola], and either a Slurpee or a snack.”* Driving was described as a means of transport for food acquisition in three distinct ways: through driving oneself, by asking a sibling for a ride, or negotiating a ride from a parent/guardian. Among those that described independent food acquisition, half indicated that they were with peers.

#### Food Spending

Adolescents exert their autonomy related to food choice through food spending. Most (*n* = 25) adolescents discussed their use of personal spending money, either provided on their own or by a parent, to make independent food purchases. Forty percent of these quotations mentioned the company of friends. While most teens described choices made when spending their own money, a few teens discussed exclusively spending money provided by parents. For example, when spending parents' money, adolescents referenced choosing a food establishment based on how much money was available to them. When spending their own money, teens indicated that they make different food choices compared to when a parent pays. Adolescents described being cost conscious; often buying cheaper items or “*something that's going to last me a long time… [such as] a big box of ramen.”* One participant described the food spending decisions this way: “*I like to go to Chipotle because I associate that with being healthier, because it's just like healthy ingredients and stuff like that. But if money is kind of pressed, I really want to go out to eat I'll just get Wendy's because it's probably the cheapest option.”* Additionally, we found that adolescents use their own funds on grocery and restaurant food items that parents refuse to purchase or on items that they do not normally eat. Adolescents indicated that their parents' level of trust in their ability to make responsible purchases may impact their food choices. On teen explained, “*Like they trust I'm not just going to blow my money on just junk food and stuff. They know that I'll pick like something healthy and something that's not as healthy.”*

#### Adolescent Influences on the Household Food Environment

In discussion of food choices in the home, adolescents described their role in shaping the food options available in the home food environment. Adolescents have various levels of involvement in grocery planning and shopping which may demonstrate elements of their eating autonomy (*n* = 27). Some adolescents described that they could contribute to a household grocery list throughout the week. Most adolescents mentioned that their grocery requests are purchased at least some of the time. One teen detailed the process of choosing grocery food items in their household:

**“…** *If you ask for more stuff then there's less chance to get it. It's basically just a timing thing- because my mom is super organized, and she has a list inside, and it's like it's sectioned off for, like, produce and cleaning supplies. And if you can get it on the list before it's taken down on Sunday, then she could get it for you. But [not] if you miss the deadline.”*

Adolescents described that parents' ultimate purchasing of requested foods are often dependent on the number, type, or cost of the items an adolescent suggests. Some adolescents explained that they are motivated to accompany parents during grocery shopping since being physically present increases the likelihood of their parent(s) purchasing an adolescents' requested food items. Still, others indicated they are not involved in selecting grocery items for their household either in advance or during a shopping trip.

#### Control Perceptions

Differing perceptions of control over their food choices was a dimension of adolescent eating autonomy that emerged in focus groups. Adolescents described having differential control over their food choice in association with eating occasions (such as dinner or snacks), time periods (such as weekends), and physical environments (such as home or restaurants). When participants described control in terms of eating occasion, about one third of adolescents referenced lunch and nearly one third mentioned breakfast as eating occasions in which they experienced higher levels of control over their food choice. Adolescents also described differences in control over their food choices based on schedule and time priorities; where reduced control was related to busy schedules and higher control was associated with periods of free time (such as the weekend). **“…** *So, like at home a little bit more freedom. Weekends, little bit more, but not during the day at school.”*

Adolescents also commented on their perceived level of control over their food choices in specific environments. The most commonly mentioned settings (one-third of participants) in which adolescents expressed having a high degree of control over their food choices were home and restaurants. However, the home setting was also commonly cited as a setting in which adolescents perceived low control (25% of participants) depending on the meal. Expressions of low control over food choices in the home were commonly associated with the dinner/family meal and having to eat what parents prepare, having “*no choice in the matter”*. When adolescents expressed having high control over food choice at restaurants, it was described mostly within the context of control over one's meal choice or choice over the restaurant itself. Only 3 adolescents expressed having high control over their food choices at school.

#### Perceived Parental Restriction

Adolescents reported that their food choice autonomy is restricted by parent(s) through various means. Teens described experiencing parental restriction through limitations on “unhealthy” food consumption, food costs, to prevent food waste, and parental over-promotion of “healthy eating.” Adolescents explained that some parents attempt to limit anything “*too unhealthy” (*such as chips and sugary items) by not purchasing them or verbally discouraging their consumption. Some expressed that they wished their food choices were less restricted by parents because they want to experience new foods inside the home and at restaurants. One teen explained, “*…I want to be able to eat stuff and trying new stuff. But sometimes she does restrict me more than she probably should.”* Some adolescents noticed differences in how family members influence the restriction of the adolescent's food choices. For example, one teen explained that their father's strictness about healthy eating results in less choice when in their company than compared to when alone or with someone else. Other adolescents described their food choices being more restricted when the whole family was home or related to dinner or the family meal.

#### Perceived Parental Awareness and Approval of Adolescents' Food Choices

Adolescents make food choices that may or may not align with their parents' wishes. Two out of three adolescents felt that their parent(s) would like them to make different food choices; adolescents cited that their parents wanted their food choices to reflect more overall balance, reduced sugar intake, and increased fruit and vegetable consumption. Adolescents also perceive a range of parental awareness regarding their food choices. About one in five adolescents referenced their parent(s) being largely aware of the food choices made when they are not present. Another 18% of adolescents mentioned moderate parental awareness and 15% perceived their parent(s) were only minimally aware of what they eat and drink on their own.

Differences in parental awareness regarding adolescents' independent food choices may be based on different levels of parental inquiry. Many adolescents stated that their parent(s) ask about their independent food choices at least some of the time. In response to parent inquiry, some adolescents openly disclose information, while others intentionally omit items that their parent(s) disapprove of, or do not disclose their food choices at all. “*No. I don't tell them. Like I'm not supposed to eat chewy things, but I still do.”* Some adolescents stated that their parent(s) never inquire about their independent food choices. In some cases, parents not asking about independent food choices was perceived as a sign of parental trust in the adolescents' decision making. Others expressed that their parent(s) approve of their independent food choices, partially because they regularly eat healthy, balanced diets in the home, so their parents are less concerned about unhealthy dietary choices made when adolescents are on their own.

## Discussion

This study examined factors adolescents perceive to influence their food choices across a range of environments. The study is unique in its investigation of adolescent food choices across an expansive range of environments, extending beyond the previous work of food choice within the home or school, and including factors across other settings (namely, stores and restaurants). In addition, adolescents were asked to consider both the eating occasion and social context, which makes this study more comprehensive than the prior work. Through this study, we identified the key features of the construct of adolescent eating autonomy toward the goal of better understanding adolescent food decision making. This is important because adolescence is a developmental period where eating habits are established that track into adulthood.

**Figure 2:**
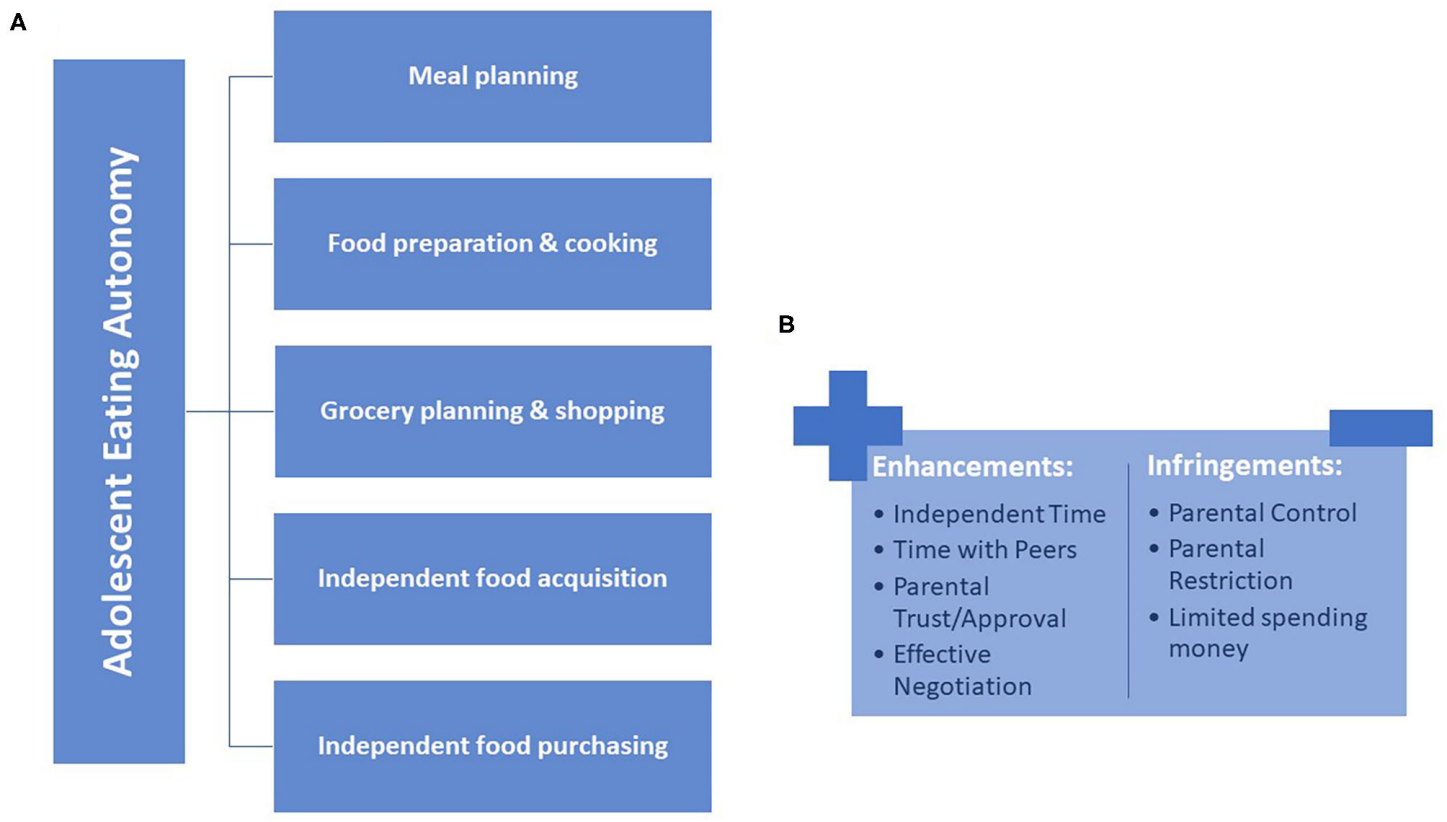
Conceptual elements of adolescent eating autonomy. **(A)** Shows the emergent autonomous food-related behaviors in which adolescents have a range of engagement. **(B)** Shows the preliminary factors that may enhance or infringe upon an adolescent's experience of eating autonomy.

These results were used to organize our initial conceptualization of adolescent eating autonomy. Figure 2A summarizes the food-related behaviors in which adolescents experience a range of autonomy. Each of the food-related behaviors included here emerged from the focus group data as an autonomous behavior-of-interest, based on more than 30% of participants commenting on their experience in each area. Figure 2B presents some of the factors, identified from the autonomy development literature and throughout these results, that may enhance or infringe upon an adolescent's experience of eating autonomy.

### Factors in Adolescent Food Choices

Research on determinants of food choice has found individual differences in the factors that are considered most influential (Köster, 2009). Under the personal food system of the food-choice process model, the top five values that have consistently emerged according to adults are taste, health, cost, convenience, and managing relationships (Connors et al., 2001). When examining adolescent food choices, our study found the most influential factors generally mirror those described in adults but differ based on environmental context. Consistent with prior qualitative work on adolescent food choices (Neumark-Sztainer et al., 1999; Sommer et al., 2014; Waddingham et al., 2018), this study found convenience and ease of preparation to be top factors in adolescent food decisions in the home, school, restaurant, and store environments. The current study replicated that schedule and time priorities were important factors in the contexts of the home and school environments (Neumark-Sztainer et al., 1999; Contento et al., 2006; Holsten et al., 2012; Sommer et al., 2014), but not in restaurants and stores. We also found that when food healthfulness was mentioned, it was typically within the context of identifying whether a chosen food was considered unhealthy or not, rather than explaining that a food was selected based on health values. This is consistent with previous work showing that a food's perceived healthiness is not a dominant factor in adolescents' food choices (Croll et al., 2001; French et al., 2001; Waddingham et al., 2018).

Previous literature often cites the taste or physical properties of food as a top factor in food choices, among both adults (Köster, 2009) and adolescents (Neumark-Sztainer et al., 1999; Contento et al., 2006; Holsten et al., 2012; Sommer et al., 2014; Bawajeeh et al., 2020). However, we found that taste was only a predominant factor in discussions about food choices at school. This mirrors work by Share and Stewart-Knox (2012) who found, through quantitative factor analysis, that taste properties were not primary determinants of Irish adolescent food choices. One reason for this is the taste of food may be more highly valued in school-based food choices, where adolescents' selections are limited to options that are perceived to be low in palatability. In contrast, at home or at a restaurant, adolescents may have more palatable options from which to choose. Our work supports the idea that adolescents have reciprocal influences on their parents' behaviors which may play a role in ensuring their home food environment regularly contains items that they approve of in terms of taste (Crosnoe and Johnson, 2011).

Cost was a prominent factor in adolescents' descriptions of food choices in restaurants and stores. To our knowledge, only one previous study has identified cost as a factor in food decisions outside the home among adolescents with obesity (Watts et al., 2015). It is possible that cost is not commonly cited as a top factor in other work about adolescent food choices because a multitude of studies have focused on adolescent food choice when the food is freely available, such as in the home or in school, and not when food selection may be influenced by making a purchase with their own funds. It is also plausible that other studies have not often seen the prominence of cost because the concepts of cost and convenience may be highly linked according to adolescents (Share and Stewart-Knox, 2012).

Eating is a social experience for adolescents (Holsten et al., 2012; Sommer et al., 2014; Waddingham et al., 2018). We found that food choices described in restaurants and stores more often co-occurred with the presence of friends compared to school and the home, which had virtually no mentions of family or friends. Furthermore, we found that adolescents were often motivated by an openness to try new foods when at restaurants (including coffee shops), where teens may feel less constrained or find trying new foods more appealing among friends. Similarly, another study investigating food choice demonstrated the “enjoyment of foods as a learning process” as a main motivator particularly in the context of dining out at restaurants, where those adolescents with food allergies felt their freedom to experience openness was limited by their dietary restrictions (Sommer et al., 2014). While our work did not find adolescents' food choices to be motivated by peer influences specifically, other studies have shown that adolescents may select foods in order to conform to those around them, to comply with gender stereotypes (Chapman and Maclean, 1993), or to meet a perceived need to select “children's food” (like chicken nuggets or a white bread sandwich) as opposed to “adults' food” (such as a salad) (Ludvigsen and Scott, 2009).

### Elements of Adolescent Eating Autonomy

This analysis identified the predominant elements comprising the construct of adolescent eating autonomy. While prior work identified concepts related to adolescent control over eating, this study is the first to synthesize and conceptualize the components of the construct. Initially, we defined adolescent eating autonomy as an adolescents' individual decision-making related to, and perceptions of control over, their food choices. This work further developed our understanding of the construct as being comprised of autonomous food-choice behaviors, for which adolescents have a range of engagement and perceived control. Furthermore, each of the autonomous food-choice behaviors may be influenced by factors which can expand, or infringe, upon adolescents' eating autonomy.

Bassett et al. (2008) suggest that adolescents' food choices are actively co-constructed through adolescents' exertions of autonomy and parental control mechanisms, which are expected to change over time (Bassett et al., 2008). For most of adolescence, we assume a parent/adolescent dynamic that is by nature interdependent. A number of food parenting practices (such as discussion, negotiation, and autonomy support) are known to play a role in building a child's self-regulation related to food (Di Pasquale and Rivolta, 2018). It is possible that autonomy-enhancing parenting practices may be related to adolescents' food choices during independent eating occasions (Reicks et al., 2020), but more controlled work needs to be done in order to distinguish adolescents' autonomous food decision making from parental influences.

Adolescents' perceptions of control over their food choice in different contexts may provide insight into their situational eating autonomy. Teens express having a range of control over their food choices based on eating occasion, time period, and environment, but the reasons for the differences in perceived control are unknown (Sommer et al., 2014). Our results mirror those of Contento et al. (2006) showing that dinner was the meal that demonstrated the greatest differences in perceived control (Contento et al., 2006). In most cases, their sense of control during dinner was constrained by the family meal in the home, unless the adolescents were engaged in other components of the meal planning, shopping, or preparation process. Availability of a variety of offerings at a given time was associated with higher perceived control over food choices in many environments. This points to the nuance of the element of control, where some may feel higher levels of control in situations where they simply have more items to choose from (such as on a restaurant menu). Others may feel more control in their immediate food choices (those based on hunger, taste, convenience) compared to choices based on more distant considerations addressed through complex behaviors (such as grocery shopping and planning).

Adolescents can demonstrate their eating autonomy through their food spending habits. This study found that adolescents often buy items that they want but a parent will not buy for them (such as popcorn at the movies or an energy drink at the grocery store). However, these autonomous spending behaviors were often associated with adolescents purchasing items viewed as treats, special occasions, items they normally don't have access to, or items outside of the household's meal plan. These findings suggest that adolescents' independent food choices are not indicative of their broader eating habits. Therefore, to better understand adolescents' food choices, we should first examine the extent and frequency for which different autonomous food-related behaviors occur.

### Strengths and Limitations

This study has many notable strengths. First, it was conducted among a diverse group of unassociated adolescents in a community location, outside of the school and home environments. Second, the research team was guided by those with extensive experience in qualitative methodology and all focus group transcripts were coded by 3 independent coders. Third, this study investigated the food choices of adolescents, which is an understudied group. Finally, the results of this work have helped to define and delineate the novel construct of adolescent eating autonomy. This work begins to address the knowledge gap related to adolescents' food choice process.

This work also has a few limitations. While the sample was racial/ethnically representative of the population under age 18 in Erie County, NY, it was still a largely white population with a higher proportion of participants were from middle- and high-income categories, compared to the local population. Also, this qualitative analysis coded independent food choices only with explicit mentions of being without parent(s); therefore, some factors may have been missed if a participant failed to provide enough context. Finally, eating contexts with a high degree of overlap (for example the dinner meal, the home environment, and parental influences) presented challenges in drawing inferences from results.

## Conclusions

Prior to this work, the factors related to adolescents' food choices had not been studied across the entirety of the diet and in a neutral setting. This study identified the main elements of adolescent eating autonomy with the aim of being able to quantify adolescents' amount of agency over their food choices. The findings from this study are being used to generate an adolescent eating autonomy scale, which is being tested, validated, and published by co-authors from this group. Assessing the level of involvement in specific autonomous eating behaviors will allow us to quantify the amount and frequency of adolescent's engagement in many food-related behaviors. We suspect that some dimensions of the eating autonomy construct may be strongly related to overall diet quality, obesity, dieting behavior, and other adolescent health outcomes.

## Data Availability Statement

The datasets presented in this article are not readily available because the data are coded, merged qualitative transcripts of verbal conversations in the Atlas.ti program. Requests to access the datasets should be directed to Amanda M. Ziegler, amz9@buffalo.edu.

## Ethics Statement

The studies involving human participants were reviewed and approved by The University at Buffalo Institutional Review Board. Written informed consent to participate in this study was provided by the participants' legal guardian/next of kin.

## Author Contributions

AZ was responsible for study design, recruitment, data collection, coding, analysis, writing, and manuscript preparation. CK was responsible for coding, analysis, writing, and manuscript preparation. TM was responsible for data collection, coding, analysis, writing, and manuscript preparation. AG, RB, and LH was responsible for recruitment, data collection, and writing. LL and JT was responsible for study design, writing, and manuscript preparation. All authors contributed to the article and approved the submitted version.

## Conflict of Interest

The authors declare that the research was conducted in the absence of any commercial or financial relationships that could be construed as a potential conflict of interest.
